# Why your smartwatch may be misleading your doctor: a cross-sectional study on the impact of mobility aids on wearable accuracy in older adults

**DOI:** 10.7717/peerj.20690

**Published:** 2026-04-15

**Authors:** Alp Özel, Elif Yakşi, Çağın Çevik, Mehmet Rahim Nazar

**Affiliations:** 1 Department of Physiotherapy and Rehabilitation, Bolu Abant Izzet Baysal University, Bolu, Turkey; 2 Department of Physical Therapy and Rehabilitation, Bolu Abant Izzet Baysal University, Bolu, Turkey; 3 Analog Devices Inc., Wilmington, MA, United States of America; 4 Department of Physiotherapy and Rehabilitation, Medipol University, Istanbul, Turkey

**Keywords:** Assistive devices, Gait, Physical activity, Wearable sensors, Ambulatory monitoring

## Abstract

**Background:**

Wrist-worn wearables like the Apple Watch are widely used to monitor physical activity in both clinical and home settings. But what happens when an middle-aged to older adults walks with a rolling walker or pushes an oxygen cart? This study shows that such devices dramatically impair step detection, leading to significant undercounting and potential clinical misinterpretation. This study aimed to evaluate the accuracy of a wrist-worn device (Apple Watch Series 8) and a waist-mounted device (Omron Walking Style IV) in middle-aged to older adults using common assistive walking aids.

**Methods:**

In a cross-sectional design, 42 community-dwelling middle-aged to older adults (aged 51–80) performed walking tasks under four assisted conditions (forearm crutch, rolling walker, oxygen trolley, and unassisted) and three treadmill speeds (1.61, 3.22, and 4.83 km/h). Step counts and distances recorded by each wearable device were compared to manual reference measures. Wilcoxon signed-rank tests and Bland–Altman analyses assessed accuracy and agreement.

**Results:**

The Apple Watch significantly underestimated step counts, particularly with a rolling walker (−36.4%, *p* < .001), and at low treadmill speed (−16.3% at 1.61 km/h). In contrast, the Omron pedometer demonstrated relatively consistent performance across most conditions, although it showed notable underestimation at the lowest walking speed (1.61 km/h). A weak correlation between arm length and Apple Watch error was observed (*r* =  − 0.38, *p* < .05).

**Conclusion:**

Wrist-worn activity monitors may be unsuitable for middle-aged to older adults using assistive devices due to undercounting. Waist-mounted devices offer more reliable step and distance tracking, supporting their use in clinical practice and home rehabilitation. Device selection and placement should be carefully considered in mobility-impaired populations.

## Introduction

Accurate assessment of physical activity is vital for monitoring health, guiding rehabilitation, and promoting functional independence in middle-aged to older adults. With global populations aging rapidly, a growing number of older individuals rely on assistive walking devices such as canes, walkers, forearm crutches, and oxygen trolleys to maintain mobility and safety (Danemayer et al., 2025). While these aids improve stability and autonomy, they also introduce gait alterations that can challenge the validity of step-counting technologies (Angelucci, Canali & Aliverti, 2023; Kriara et al., 2025).

Wearable activity monitors, including wrist-worn smartwatches and waist-mounted pedometers, are commonly used in both clinical and home settings to quantify physical activity (Szeto, Arnold & Maher, 2024). Their portability and ability to provide real-time feedback have made them popular tools in adapted physical activity (APA), telerehabilitation, and long-term self-monitoring programs (Arntz et al., 2023). However, existing research indicates that their accuracy can be significantly influenced by walking conditions, particularly in individuals with altered gait patterns or limited arm swing (Bianchini et al., 2023; Pan & Wei, 2024). For example, wrist-based devices like the Apple Watch have demonstrated substantial underestimation of step counts in populations walking slowly or using mobility aids (Shiwani et al., 2023). In contrast, waist-mounted devices such as the Omron pedometer have shown more consistent performance in middle-aged to older adults and clinical populations (Sun et al., 2025; Vavasour et al., 2023).

Despite the increasing adoption of wearables in adapted physical activity (APA) and clinical practice, previous studies have mainly focused on general step-count accuracy in older adults, while limited evidence exists on how specific types of assistive walking aids such as rollators, forearm crutches, or oxygen trolleys influence measurement validity (Abdul Jabbar et al., 2023; Keogh et al., 2021; Moore et al., 2021; Schmidle et al., 2023). This gap in the literature presents a challenge for practitioners who rely on these devices to monitor physical activity and guide interventions. Without validation in the presence of mobility aids, wearable-derived metrics may lead to misinterpretation of physical capacity or treatment response.

This cross-sectional study aimed to evaluate the impact of commonly used assistive walking aids on the accuracy of step count and distance measurements recorded by two commercially available wearable devices: the Apple Watch Series 8 (wrist-worn) and the Omron Walking Style IV (waist-mounted), in community-dwelling middle-aged to older adults.

### Primary hypothesis

The use of assistive walking devices significantly impairs the accuracy of wrist-worn step counters (*e.g.*, Apple Watch) compared to waist-mounted devices (*e.g.*, Omron). By addressing the interaction between gait adaptations and wearable sensor performance, this study contributes to the growing body of evidence required for informed device selection, accurate interpretation of wearable-derived data, and future development of wearable technologies tailored to APA contexts.

## Materials & Methods

### Study design and setting

This cross-sectional validation study was conducted between December 2023 and June 2024 in a controlled clinical environment at the Bolu Abant Izzet Baysal University, Physiotherapy and Rehabilitation Department. The study protocol received ethical approval from the Non-Interventional Clinical Research Ethics Committee of Bolu Abant Izzet Baysal University (Approval No: 2023/334, Date: 7/11/2023), and written informed consent was obtained from all participants in accordance with the Declaration of Helsinki.

### Participants

The study included 42 community-dwelling adults aged 50 to 81 years (45% female). This broad age range was intentionally selected to represent different functional stages of aging to capture age-related gait variability and its potential influence on wearable-device accuracy. Such inclusion reflects real-world clinical diversity and enhances the translational relevance of the findings for adaptive physical-activity and geriatric-rehabilitation contexts.

**Figure 1:**
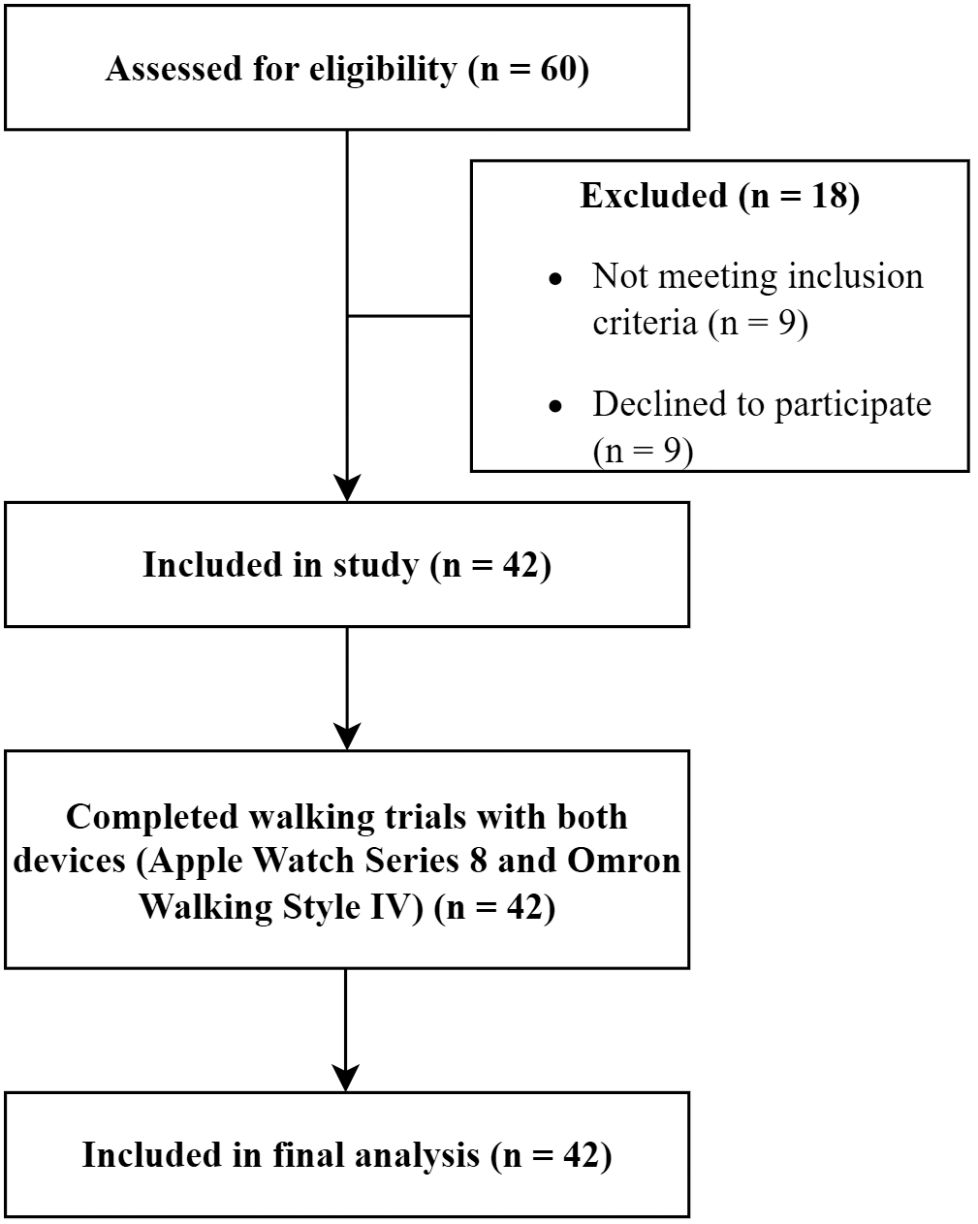
Flow diagram of participant recruitment. A total of 60 older adults were assessed for eligibility. Of these, 18 were excluded (nine did not meet inclusion criteria; nine declined participation). Forty-two participants were enrolled, completed walking trials with both devices (Apple Watch Series 8 and Omron Walking Style IV), and were included in the final analysis.

All participants were community-dwelling, functionally independent middle-aged to older adults who were able to complete standardized walking tasks independently. Based on self-reported health information, 16 participants (38%) had controlled hypertension, nine (21%) had type 2 diabetes, and seven (17%) reported mild knee osteoarthritis. No participant had any cardiopulmonary condition that could affect gait symmetry. Eligibility criteria comprised the ability to ambulate independently, with or without the use of an assistive device, and absence of acute neurological or orthopedic disorders. Demographic and anthropometric variables collected included age, sex, height, weight, body mass index (BMI), and arm length. A flow diagram summarizing participant recruitment and testing procedures is presented in Fig. 1.

### Walking conditions and protocol

Participants completed the six-minute walk test (6MWT) under four separate conditions: (1) unassisted walking, (2) walking with a forearm crutch, (3) walking with a rolling walker, and (4) walking while pushing an oxygen cylinder trolley. Additionally, participants walked on a treadmill at three self-paced speeds (1.61 km/h, 3.22 km/h, and 4.83 km/h) to assess device performance at varying walking intensities. Each treadmill walking trial was performed for a fixed duration of 2 min at each speed (1.61 km/h, 3.22 km/h, and 4.83 km/h), allowing consistent data collection across all participants. During assisted walking conditions, a soft knee braces (neoprene sleeve) was worn on the dominant leg to simulate mild joint restriction and promote consistent gait adaptations. To minimize potential order effects such as fatigue or learning, the sequence of walking conditions was randomized using a computer-generated randomization list. This sequence was generated prior to data collection using a random number generator in Microsoft Excel. Each participant was assigned a unique random sequence that determined the order in which the four walking conditions (unassisted, forearm crutch, rolling walker, oxygen trolley) and three treadmill speeds (1.61 km/h, 3.22 km/h, and 4.83 km/h) were performed. The assigned sequences were placed in sealed envelopes and opened immediately before testing to ensure allocation concealment. Each test was separated by a minimum of 10 min of seated rest. All procedures were conducted according to the European Respiratory Society (ERS)/American Thoracic Society (ATS) guidelines for the 6MWT (Holl et al., 2014).

### Devices and measurement

Step count and walking distance were simultaneously measured using two commercially available wearable devices: Apple Watch Series 8 (Apple Inc., Cupertino, CA, USA), worn on the dominant wrist; Omron Walking Style IV (Omron Healthcare Co., Ltd., Kyoto, Japan), worn on the dominant-side waist. The selection of wearable devices was based on their widespread clinical and consumer use, prior validation evidence, and technical suitability for step-counting accuracy studies (Doherty et al., 2024). The Apple Watch Series 8 was chosen as a representative wrist-worn consumer-grade device that integrates a triaxial accelerometer and gyroscope. It has been extensively validated for activity monitoring and step detection in older and clinical populations (Spartano et al., 2024). The Omron Walking Style IV was selected as a waist-mounted comparator due to its proven reliability, triaxial accelerometer sensor, and consistent performance in rehabilitation and geriatric research settings (Holbrook, Barreira & Kang, 2009). Device placement followed manufacturer guidelines: the Apple Watch was worn on the dominant wrist, while the Omron pedometer was attached to the waistband at the dominant side.

Manual step counts were recorded using a tally counter by an experienced physiotherapist and served as the reference (criterion) measure. Walking distance was determined by multiplying the number of completed laps by the standardized walkway length (30 m).

### Outcome measures

• Step count error: Device-recorded steps *vs.* manual count.
• Distance error: Device-recorded distance *vs.* calculated true distance.
• Percentage error was calculated using the formula:

\begin{eqnarray*}Percentage~Error= \frac{(\mathrm{Device}-\mathrm{Reference})}{Reference} \ast 100. \end{eqnarray*}

### Statistical analysis

*A priori* sample size calculation was performed using G*Power (Version 3.1.9.7; Heinrich-Heine-Universität Düsseldorf, Düsseldorf, Germany). Based on prior research examining the accuracy of wearable step counters in mobility-impaired populations, an expected medium effect size (*d* = 0.5) was assumed (Holubová et al., 2022). With a significance level of α = 0.05 and a statistical power of 80% (1–β = 0.80), the required sample size for detecting significant differences in device accuracy under repeated measures design was calculated to be 34 participants. The final sample of 42 participants thus provided sufficient power to test the primary hypothesis.

All analyses were performed using IBM SPSS Statistics (Version 20.0; IBM Corp., Armonk, NY, USA). The normality of continuous variables was assessed using the Shapiro–Wilk test. Descriptive statistics were reported as mean ± standard deviation (SD) for normally distributed variables and median with interquartile range (IQR) for non-normally distributed ones. Categorical variables were summarized as frequencies and percentages. Since the primary outcome variables did not meet the assumption of normality, the Wilcoxon signed-rank test was used to compare step count and distance measurements between the wearable devices and the manual reference. The Wilcoxon signed-rank test was applied not as a measure of reliability or validity, but to identify potential systematic bias between devices under each walking condition, consistent with a previous validation study employing nonparametric comparisons (Kooiman et al., 2015). The reported *z* values represent standardized test statistics derived from the Wilcoxon test, indicating the direction and magnitude of paired differences between devices. Effect sizes (r) were reported to indicate the magnitude of differences between devices in the nonparametric analysis. Agreement between the devices and the reference method was further examined using Bland–Altman plots to identify systematic bias. To explore potential associations between device error rates (for both step count and distance) and participant characteristics such as age, BMI, and arm length, Spearman’s rank correlation coefficients were calculated. A two-tailed *p*-value less than 0.05 was considered statistically significant for all analyses. No missing data were observed.

## Results

### Participant characteristics

A total of 42 middle-aged to older adults (23 males, 19 females) aged between 51 and 80 years (*M* = 60.6, SD = 5.8) participated in the study. The average BMI was 27.5 ± 4.1 kg/m^2^, indicating that most participants were classified as overweight. The median height was 167.5 cm (IQR = 158.5–175.0), and the median arm length was 50.0 cm (IQR = 48.0–52.8). Participant characteristics are summarized in Table 1.

**Table 1:** Participant characteristics. Descriptive statistics for age, weight, height, BMI, and arm length. Values are presented as mean (standard deviation) or median (interquartile range), depending on data distribution.

Variable	M (SD)	Mdn (IQR)
Age (years)	N/A	59.0 (57.2–61.8)
Weight (kg)	76.3 (12.7)	N/A
Height (cm)	N/A	167.5 (158.5–175.0)
BMI (kg/m^2^)	27.5 (4.1)	N/A
Arm length (cm)	N/A	50.0 (48.0–52.8)

**Notes.**

M mean SD standard deviation N/A Not applicable Mdn median IQR interquartile range

### Step count accuracy across devices and conditions

Across all walking conditions, the Apple Watch significantly underestimated both step count and walking distance, while the Omron pedometer showed smaller and often statistically non-significant deviations. As shown in Table 2, the Apple Watch underestimated step count by a median of -39.5 steps (IQR: −113.5 to −2.0), while the Omron pedometer showed a small overestimation (*M* = 11.0 ± 28.3 steps, *p* < .001). Regarding distance, the Apple Watch underestimated by −143.6 ± 135.2 m (*p* < .001), and the Omron by −119.6 ± 133.9 m (*p* = .080).

**Table 2:** Step count and distance measurement differences compared to manual measurement. Descriptive statistics for step count and distance discrepancies recorded by Apple and Omron devices in comparison to manual measurements. Values are reported as mean (SD) and median (IQR) where appropriate. Positive values reflect overestimation, while negative values reflect underestimation relative to manual reference.

Variable	M (SD)	Mdn (IQR)	*p*-value
Step Count Difference (Apple)	N/A	−39.5 (−113.5 to −2.0)	.131
Step Count Difference (Omron)	11.0 (28.3)	11.0 (−6.8 to 31.2)	<.001^a^
Distance Difference (Apple)	−143.6 (135.2)	N/A	<.001
Distance Difference (Omron)	−119.6 (133.9)	N/A	.080^a^

**Notes.**

M mean SD standard deviation N/A not applicable

Values are presented as mean (standard deviation) and median (interquartile range). Positive values indicate overestimation and negative values indicate underestimation compared to manual measurements. Error rates are calculated as the percentage difference from manual step counts.

a Wilcoxon signed-rank test comparing device measurements to manual reference.

### Treadmill speed-based differences

**Figure 2:**
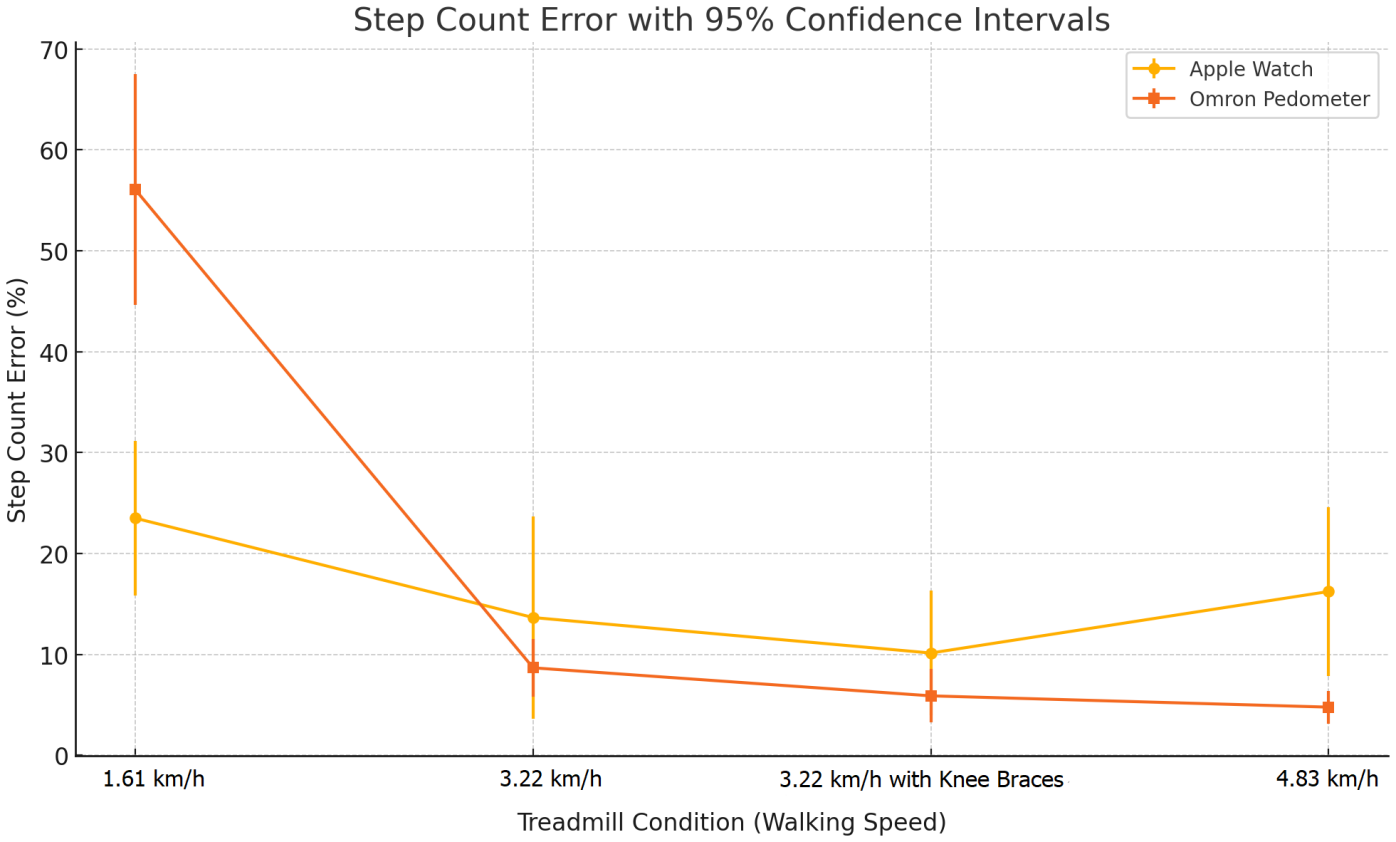
Step count error across treadmill conditions for Apple Watch and Omron Pedometer. Mean step count error (%) with 95% confidence intervals is plotted for both the Apple Watch Series 8 and Omron Walking Style IV across four treadmill conditions: 1 mph, 2 mph, 2 mph with simulated gait restriction (knee braces), and 3 mph. The Apple Watch showed greater error at lower speeds and under gait-altering conditions, while the Omron pedometer maintained relatively stable and lower error rates throughout.

**Figure 3:**
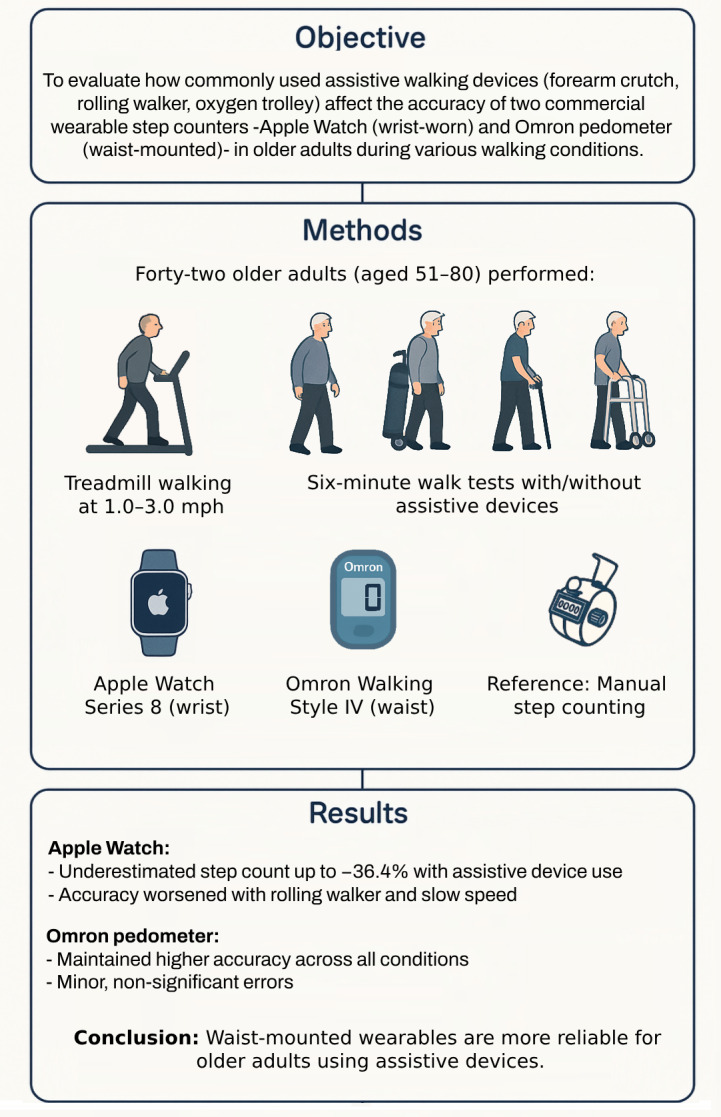
Impact of assistive devices on wearable step counter accuracy in older adults. A summary of the study’s objective, methodology, and key findings. Forty-two older adults (aged 51–80) performed treadmill walking and six-minute walk tests with and without assistive devices (*e.g.*, forearm crutch, rolling walker, oxygen trolley). Step counts recorded by the Apple Watch Series 8 (wrist-worn) and Omron Walking Style IV (waist-mounted) were compared to manual step counting. Results showed that the Apple Watch significantly underestimated steps up to −36.4% especially with rolling walkers and at low speeds. In contrast, the Omron pedometer maintained higher accuracy across all walking conditions. The findings support the use of waist-mounted wearables for reliable activity monitoring in mobility-impaired older adults.

Step count accuracy varied significantly with treadmill speed, as detailed in Table 3. At 1.61 km/h, the Apple Watch showed a mean error of −16.3% (*p* = .001), and the Omron exhibited −44.8% error (*p* = .001). However, at 3.22 km/h, the devices showed near-zero error. These findings are further illustrated in Fig. 2, which displays mean step count error with 95% confidence intervals across treadmill speeds. An overview of the study design and the main findings is summarized in Fig. 3.

**Table 3:** Device step count and percentage error compared to manual counting by treadmill speed. Step count performance of Apple Watch and Omron devices compared to manual (Tally Counter) measurements across different treadmill walking speeds. Results are presented as mean (SD), median, and range. Percentage error indicates the relative deviation from manual counting. Statistical comparisons (Wilcoxon signed-rank test with Bonferroni correction) were applied to device measurements; no statistical tests were conducted for manual reference values.

Walking speed (km/h)	Device	Mean step count (SD)	Median	Range	Mean error (%)	*z*-score	*p*-value (Bonferroni)^*^	Effect size (*r*)
**1.61**	Apple Watch	152.98 (66.26)	158	9-298	−16.3%	−3.45	.001	0.53
	Omron	100.93 (99.65)	83	0-296	−44.8%	−4.57	.001	0.70
	Tally Counter	182.86 (32.80)	174	139–254	0.0%	N/A	N/A	N/A
**3.22**	Apple Watch	231.43 (83.77)	221	0–595	−0.1%	−0.17	.999	0.02
	Omron	240.90 (40.38)	239	134–329	+3.9%	−2.38	.051	0.36
	Tally Counter	231.76 (29.29)	226	185–303	0.0%	N/A	N/A	N/A
**3.22** **+ Knee Braces**	Apple Watch	216.57 (53.36)	224	0–326	−7.6%	−1.70	.264	0.26
	Omron	229.38 (29.53)	229	155–296	−2.1%	−0.08	.999	0.01
	Tally Counter	234.31 (30.92)	188	188–355	0.0%	N/A	N/A	N/A
**4.83**	Apple Watch	260.63 (84.59)	258	19–524	−6.9%	−2.06	.115	0.32
	Omron	276.07 (44.97)	268	232–501	−1.4%	−0.93	.999	0.14
	Tally Counter	279.90 (48.41)	263	229–500	0.0%	N/A	N/A	N/A

**Notes.**

M mean SD standard deviation N/A not applicable km/h kilometers per hour

Statistical comparisons were not performed for manual reference values (Tally Counter). Tally Counter used as gold standard.

*p*-values based on Wilcoxon signed-rank test;

* Bonferroni correction applied for multiple comparisons.

*p* < .05 considered statistically significant.

### Performance during 6MWT

Device performance also varied significantly based on assistive device use during the 6MWT, as presented in Table 4. The largest error was seen in the Apple Watch during walking with a rolling walker, with a −36.4% underestimation (*p* = .001). In contrast, the Omron consistently showed minor overestimations across conditions (ranging from +2.2% to +5.3%), most of which were not statistically significant after Bonferroni correction.

**Table 4:** Device step count, percentage error, and distribution during the 6MWT. Step count performance and error rates of Apple Watch and Omron devices compared to manual counting (Tally Counter) across four walking conditions during the 6MWT: without assistive device, with oxygen cylinder trolley, with rolling walker, and with forearm crutch. Values are presented as mean (SD), median, and range. Error percentages indicate overestimation (+) or underestimation (−) relative to manual counts. Statistical comparisons were performed using the Wilcoxon signed-rank test with Bonferroni correction.

Walking condition	Device	M (SD)	Mdn	Range	Error (%)	z-score	p-value (Bonferroni)^a^	Effect size (r)
6MWT^b^ (no assistive device)	Apple Watch	634.26 (68.21)	647	455–751	+1.9	−1.52	.394	0.24
	Omron	649.26 (93.77)	670	251-755	+4.4	−3.71	.001	0.57
	Tally Counter	622.17 (80.04)	635	401–722	0.0	N/A	N/A	N/A
6MWT + oxygen cylinder trolley	Apple Watch	625.05 (100.09)	635	317–807	+0.1	−0.77	.999	0.12
	Omron	657.64 (78.38)	670	404–804	+5.3	−3.70	.001	0.57
	Tally Counter	624.60 (72.18)	635	401–726	0.0	N/A	N/A	N/A
6MWT + rolling walker	Apple Watch	398.02 (186.33)	445	0–800	−36.4	−5.27	.001	0.81
	Omron	652.71 (92.90)	664	353–851	+4.3	−2.69	.021	0.42
	Tally Counter	626.12 (70.03)	632	426–746	0.0	N/A	N/A	N/A
6MWT + forearm crutch	Apple Watch	567.43 (96.52)	583	277–804	−9.5	−3.90	.001	0.60
	Omron	641.14 (81.36)	634	405–873	+2.2	−1.99	.140	0.31
	Tally Counter	627.36 (64.94)	621	477-805	0.0	N/A	N/A	N/A

**Notes.**

M mean SD standard deviation N/A not applicable Mdn median

Error rates are relative to manual step counts using a tally counter. Negative values indicate underestimation, positive values indicate overestimation.

a Comparisons were conducted using the Wilcoxon signed-rank test. Bonferroni correction applied for multiple comparisons.

b 6MWT: Six-Minute Walk Test.

### Device-to-device comparison

Direct comparisons between the Apple Watch and Omron across all walking conditions are summarized in Table 5. Statistically significant differences in step count estimates were observed in most conditions, especially with assistive device use. For example, during walking with a rolling walker, a significant difference was found (*z* = −5.47, *p* < .001, *r* = .84), indicating higher accuracy in Omron. However, no significant difference was found during 3.22 km/h treadmill walking (*p* = .228), suggesting improved agreement at moderate speeds.

**Table 5:** Wilcoxon signed-rank test comparison between Apple Watch Series 8 and Omron Walking Style IV under each walking condition. Statistical comparisons of step counts recorded by the Apple Watch Series 8 and Omron Walking Style IV across various walking conditions, including treadmill walking at different speeds and the Six-Minute Walk Test (6MWT) with or without assistive devices. Reported values include z-scores, uncorrected and Bonferroni-corrected p-values, and effect sizes.

Walking condition	*z* score	*p* value (uncorrected)^a^	*p* Value (Bonferroni)^b^	Effect size (*r*)
Treadmill 1.61 km/h	−3.37	.001	.001	0.52
Treadmill 3.22 km/h	−1.77	.077	.228	0.27
Treadmill 3.22 km/h + knee braces	−2.15	.032	.095	0.33
Treadmill 4.83 km/h	−2.44	.015	.040	0.38
6MWT (unassisted)	−3.05	.002	.007	0.47
6MWT + oxygen trolley	−2.46	.014	.038	0.38
6MWT + rolling walker	−5.47	.001	.001	0.84
6MWT + forearm crutch	−4.55	.001	.001	0.70

**Notes.**

a *p* values are based on Wilcoxon signed-rank tests comparing step counts between Apple Watch Series 8 and Omron Walking Style IV under each walking condition.

b Bonferroni correction was applied for multiple comparisons.

*p* < .05 was considered statistically significant.

### Error distributions and visual comparisons

Grouped bar charts visualizing step count distributions across devices and walking conditions are provided in Fig. S1. Bland–Altman plots illustrating agreement in step counts and distances are presented in Figs. S2, S3, respectively.

### Correlates of device error

Correlations between participant characteristics and device error rates are presented in Table S1. No significant correlations were found for age, BMI, or sex. However, a weak but significant correlation was observed between arm length and Apple Watch step count error during assisted walking (*r* = −0.38, *p* < .05).

Table S2 shows strong positive correlations between step count error and distance error for both devices (Apple Watch: *ρ* = .69; Omron: *ρ* = .78, both *p* < .01), indicating that distance inaccuracies likely stem from step detection errors.

## Discussion

This study demonstrated that the use of assistive walking devices significantly affects the accuracy of wrist-worn wearable activity monitors in middle-aged to older adults, with the Apple Watch consistently underestimating step counts and walking distances across various conditions. In contrast, the waist-mounted Omron pedometer maintained greater measurement stability, aligning with previous findings in clinical populations where gait alterations or restricted arm movements were present. According to previous validation studies, a step count percentage error within ±10% is generally considered acceptable for wearable activity monitors in controlled walking conditions (Kooiman et al., 2015; Feehan et al., 2018; Straiton et al., 2018). In our study, the Apple Watch demonstrated a mean error of −16.3% at 1.61 km/h, which exceeds this commonly accepted threshold, indicating limited accuracy at lower walking speeds.

The most notable discrepancy occurred during the 6MWT with a rolling walker, where the Apple Watch underestimated step counts by an average of 36.4%, while the Omron remained within a ±5% error margin. This substantial difference likely results from restricted arm movement during assistive ambulation, which limits accelerometer-based signal detection on wrist-worn devices. These findings are consistent with previous reports indicating reduced accuracy of wrist-worn wearables in clinical populations with altered gait or upper limb limitations (Alinia et al., 2017; Case et al., 2015; Kooner et al., 2021; Tedesco, Barton & O’Flynn, 2017). However, user experience factors such as device comfort, usability, and technological literacy were not addressed in this study, even though they are essential for long-term adherence and real-world implementation in aging populations.

The treadmill-based phase of this study further reinforced this pattern. As shown in Table 3 and Fig. 2, the Apple Watch demonstrated significant measurement error at 1.61 km/h, with accuracy improving at 3.22 km/h and above. The Omron pedometer demonstrated more consistent performance compared to the Apple Watch across walking speeds; however, it still showed notable underestimation at lower speeds (*e.g.*, −44.8% at 1.61 km/h). These findings suggest that gait speed and upper limb involvement play a critical role in the accuracy of wearable devices, particularly those worn on the wrist. These findings are consistent with prior studies indicating that slower gait speeds and reduced arm swing can impair the accuracy of wrist-worn devices (Caroppo et al., 2025; Chan, Lord & Brodie, 2024).

Importantly, direct device-to-device comparisons (Table 5) revealed statistically significant differences in nearly all walking conditions except for moderate-speed treadmill walking. These observations support the broader literature suggesting that waist-mounted monitors are more reliable in populations with impaired or assisted mobility, including individuals with COPD, Parkinson’s disease, and post-stroke hemiparesis (Demeyer et al., 2014; Garcia et al., 2021; Storm, Heller & Mazzà, 2015).

Distance estimation was also systematically underestimated across devices, with greater error observed in the Apple Watch. This is likely due to inconsistencies in stride length and gait variability, which are common in older adults and individuals using assistive devices. Since consumer-grade wearables estimate distance by multiplying step count by a fixed stride length, any deviation from normative walking mechanics leads to compounded errors (Tedesco, Barton & O’Flynn, 2017; Singh et al., 2024).

Interestingly, no significant correlations were found between device error and participant characteristics such as age, sex, or BMI (Table S1), suggesting that general anthropometric traits may play a lesser role in wearable performance compared to walking conditions and assistive device use.

However, a weak but statistically significant negative correlation was observed between arm length and Apple Watch step count error during assisted ambulation (*r* = −0.38, *p* < .05). This finding indicates that individual limb biomechanics such as arm length can influence wrist-based accelerometer accuracy, especially when arm swing is restricted by assistive devices. Such hidden biases may disproportionately affect individuals with shorter limbs or altered upper limb kinematics, and warrant further investigation in future validation studies.

In contrast, both devices exhibited strong positive correlations between step count and distance errors (Table S2), reinforcing that distance inaccuracies largely stem from flawed step detection rather than stride length estimation alone.

From an adaptive physical activity perspective, these findings are clinically significant. Wearables are increasingly used to monitor physical activity, guide personalized exercise prescriptions, and motivate older adults in home-based rehabilitation or telerehabilitation programs (Arntz et al., 2023; Androutsou et al., 2020). However, step-based feedback becomes misleading if the accuracy of the data is compromised by assistive device use. Such inaccuracies may lead to underestimation of mobility levels, reduced motivation, or inappropriate clinical decision-making in adaptive interventions (Hosseini et al., 2023; Schumann & Doherty, 2024).

The superior performance of the waist-mounted Omron device highlights the importance of selecting appropriate device types and placement strategies in adaptive physical activity settings. Clinicians and researchers should not assume that all wearables perform equally across movement contexts (Puterman et al., 2021). Instead, device validation must be context-specific, particularly when applied in populations with altered gait or mobility limitations (Keogh et al., 2021).

Finally, beyond technical performance, the usability and acceptability of these devices in older adults must be considered. Age-related sensory, cognitive, or dexterity limitations may affect how these individuals interact with wearable technologies (Dixon, Anderson & Lazar, 2022). Future validation studies should incorporate user-centered outcomes such as device comfort, ease of use, and adherence, in addition to objective accuracy metrics.

While our findings highlight the limitations of current consumer-grade wearables, they also open a path toward smarter and more inclusive solutions. Future research should explore adaptive signal processing techniques such as dynamic thresholding, gait pattern recognition, or sensor fusion using IMUs or smart insoles to correct for the signal artifacts introduced by assistive device use. These approaches may lead to the development of wearable algorithms tailored for geriatric and mobility-impaired populations, ensuring more reliable data for clinicians and caregivers.

### Clinical implications

Wearable activity monitors are increasingly utilized in geriatric rehabilitation, home-based care, and telehealth interventions to guide clinical decisions and track functional progress. However, this study highlights a critical blind spot: wrist-worn devices, such as the Apple Watch, significantly underestimate step counts when assistive walking aids are used, particularly due to restricted arm movement and altered gait dynamics.

Such undercounting may mislead clinicians into underprescribing exercise, misclassifying patients as sedentary, or misinterpreting rehabilitation outcomes, ultimately compromising care quality. These inaccuracies can also discourage patients who rely on wearables for self-monitoring, potentially reducing motivation and adherence. Importantly, clinicians should also consider common comorbidities that influence gait and device accuracy. Importantly, clinicians should also consider common comorbidities that substantially influence gait mechanics and wearable device accuracy. Conditions such as knee osteoarthritis, type 2 diabetes, and cardiovascular disease induce characteristic alterations in gait dynamics including reduced stride length, slower cadence, and diminished arm swing all of which may compromise the sensitivity of wrist-worn accelerometers (Kobsar et al., 2020; Laffi et al., 2025; Zeng et al., 2024).

Older adults with knee osteoarthritis often exhibit increased gait asymmetry and reduced angular velocity of arm movement due to pain-related adaptations (Bacon et al., 2024). Similarly, individuals with type 2 diabetes or diabetic peripheral neuropathy show slower walking speeds, prolonged stance times, and greater step-to-step variability, leading to systematic undercounting in activity trackers (Hulshof et al., 2024). Moreover, cardiovascular conditions such as chronic heart failure are associated with reduced gait velocity and limited functional endurance, which further exacerbate device inaccuracy, particularly in wrist-worn sensors that rely on rhythmic acceleration patterns (Pan & Wei, 2024; Pepera et al., 2012).

Our findings support the clinical superiority of waist-mounted devices like the Omron pedometer, which showed consistent performance across assistive device conditions. Therefore, clinical guidelines and remote monitoring protocols should explicitly specify device type and placement in mobility-impaired populations.

Furthermore, manufacturers and researchers should invest in adaptive algorithms capable of detecting atypical gait patterns, such as those involving rolling walkers or crutches. Until such solutions are implemented, clinicians must interpret wearable-derived metrics with caution and rely on validated technologies tailored to the patient’s mobility context.

### Limitations

This study has several limitations that should be acknowledged. First, although the sample size (*N* = 42) was sufficient to detect statistically significant differences across conditions, its modest size and single-center recruitment may limit generalizability. Future multicenter studies with larger and more diverse samples are warranted to confirm these findings across broader populations of middle-aged to older adults with varying mobility impairments.

Second, all walking trials were conducted in a controlled indoor environment with flat, obstacle-free surfaces and standardized lighting and supervision. While this design minimized confounding variables and enhanced internal validity, it may not fully represent real-world conditions such as uneven terrain, environmental distractions, or concurrent cognitive and functional tasks. Therefore, ecological validity may be limited.

Third, the study focused on only two commercially available wearable devices, both commonly used in clinical and consumer settings. Although these choices reflect real-world usage, the exclusion of other wearable technologies such as thigh-worn IMUs, smart insoles, or newer-generation wristbands limits the scope of comparison.

Fourth, although we examined potential correlates of measurement error such as age, sex, BMI, and arm length, other factors such as walking cadence, joint range of motion, upper limb dominance, and individual gait compensations were not included. The sample included relatively healthy and mobile community-dwelling middle-aged to older adults. The results may not generalize to those with advanced mobility impairments or institutionalized populations. These may influence device accuracy in subtle but important ways. Additionally, it should be acknowledged that none of the participants were habitual walking-aid users, which limits the generalizability of the findings to populations with long-term assistive device dependence.

Finally, we did not assess long-term wearability, user adherence, or perceived usability of the devices. These human factors are critical for practical application in home-based monitoring and telerehabilitation contexts. We did not assess user comfort, usability, or engagement with the devices, which are critical for sustained use in geriatric populations. Future studies should integrate both quantitative accuracy and qualitative feedback to support real-world implementation.

Despite its limitations, this study has several notable strengths. To our knowledge, it is one of the few investigations to systematically evaluate the accuracy of wearable step counters across multiple assistive device conditions in a controlled setting. The inclusion of both wrist- and waist-mounted devices allows for a direct comparison of sensor placement effects, which is highly relevant for clinical decision-making. Additionally, the use of standardized protocols such as the 6MWT, the incorporation of multiple speed conditions, and the use of manual counting as a reference enhance the internal validity and methodological rigor of the study. The inclusion of correlation analyses further provides mechanistic insight into potential sources of measurement error, such as arm length and walking speed.

## Conclusions

In conclusion, this study highlights the impact of assistive device use on the measurement accuracy of wearable step counters in middle-aged to older adults. Wrist-worn devices such as the Apple Watch demonstrate substantial underestimation during assisted ambulation, particularly when arm movement is restricted. Conversely, waist-mounted pedometers like the Omron offer more consistent performance across conditions. These findings reinforce the importance of matching device placement to movement characteristics in clinical and home-based adapted physical activity programs. Clinicians, researchers, and rehabilitation professionals should interpret wearable-derived data cautiously and prioritize technologies validated in mobility-impaired populations. Existing wrist-worn activity monitors without specific adjustment for walking-aid usage may therefore be unsuitable for accurate step-count assessment in such individuals. Clinicians, researchers, and rehabilitation professionals should exercise caution when relying on wrist-worn devices in populations with mobility impairments, particularly in home-based care, telerehabilitation, or adaptive exercise programs. To enhance the clinical utility of wearable technologies in such populations, future research should focus on the development of adaptive algorithms capable of accommodating atypical gait patterns and assistive device use.

## Supplemental Information

10.7717/peerj.20690/supp-1 Supplemental Information 1 Step count distributions across walking conditions by measurement method Compares step counts recorded manually (gray), by the Apple Watch Series 8 (blue), and the Omron Walking Style IV pedometer (green) across various walking conditions. These include treadmill (TM) walking at 1–3 mph, TM with knee brace, and six-minute walk tests (6MWT) with and without assistive devices (oxygen trolley, walker, and forearm crutch). The Apple Watch consistently underestimated steps during assisted walking, especially with the walker, while the Omron pedometer remained closer to manual counts across all conditions.

10.7717/peerj.20690/supp-2 Supplemental Information 2 Bland–Altman Plot comparing step counts: Apple Watch vs Manual tally counter Illustrates the agreement between step counts recorded by the Apple Watch Series 8 and manual step counts (clicker method). The x-axis represents the mean step count between the two methods, while the y-axis shows the difference (Apple –Manual). The red dashed line indicates the mean bias, suggesting a systematic underestimation by the Apple Watch. Dotted lines represent the 95% limits of agreement. The plot demonstrates moderate variability and consistent undercounting by the Apple Watch across the step count range.

10.7717/peerj.20690/supp-3 Supplemental Information 3 Bland–Altman Plot comparing distance estimates: Apple Watch vs Manual reference Compares distance measurements obtained from the Apple Watch Series 8 with manually calculated reference values based on actual walking distance. The x-axis shows the mean distance (in meters) between the two methods, while the y-axis displays the difference (Apple –Manual). The red dashed line represents the mean bias, indicating a consistent underestimation of walking distance by the Apple Watch. Dotted lines indicate the 95% limits of agreement. The plot reveals increased variability in distance estimates at higher walking distances and confirms the Apple Watch’s reduced accuracy in mobility-impaired conditions.

10.7717/peerj.20690/supp-4 Supplemental Information 4 Distance measurements, distribution, and percentage error of wearable devices compared to manual measurement across all walking conditions

10.7717/peerj.20690/supp-5 Supplemental Information 5 Spearman correlation matrix between participant characteristics and device error rates

10.7717/peerj.20690/supp-6 Supplemental Information 6 STROBE Checklist

## Additional Information and Declarations

### Data Availability

The following information was supplied regarding data availability: The datasets are available at Zenodo: OZEL, A. (2025). Raw Dataset: Impact of Assistive Walking Aids on Wearable Accuracy in Aging Populations [Data set]. Zenodo. https://doi.org/10.5281/zenodo.15789428.

## References

[ref-1] Abdul Jabbar K, Mc Ardle R, Lord S, Kerse N, Del Din S, Teh R (2023). Physical activity in community-dwelling older adults: which real-world accelerometry measures are robust? A systematic review. Sensors.

[ref-2] Alinia P, Cain C, Fallahzadeh R, Shahrokni A, Cook D, Ghasemzadeh H (2017). How accurate is your activity tracker? A comparative study of step counts in low-intensity physical activities. JMIR Mhealth Uhealth.

[ref-3] Androutsou T, Kouris I, Anastasiou A, Pavlopoulos S, Mostajeran F, Bamiou DE, Genna GJ, Costafreda SG, Koutsouris D (2020). A smartphone application designed to engage the elderly in home-based rehabilitation. Frontiers in Digital Health.

[ref-4] Angelucci A, Canali S, Aliverti A (2023). Digital technologies for step counting: between promises of reliability and risks of reductionism. Front Digit Health.

[ref-5] Arntz A, Weber F, Handgraaf M, Lällä K, Korniloff K, Murtonen KP, Chichaeva J, Kidritsch A, Heller M, Sakellari E, Athanasopoulou C, Lagiou A, Tzonichaki I, Salinas-Bueno I, Martínez-Bueso P, Velasco-Roldán O, Schulz RJ, Grüneberg C (2023). Technologies in home-based digital rehabilitation: scoping review. JMIR Rehabilitation and Assistive Technologies.

[ref-6] Bacon KL, Felson DT, Jafarzadeh SR, Kolachalama VB, Hausdorff JM, Gazit E, Stefanik JJ, Corrigan P, Segal NA, Lewis CE, Nevitt MC, Kumar D (2024). Gait alterations and association with worsening knee pain and physical function: a machine learning approach with wearable sensors in the multicenter osteoarthritis study. Arthritis Care & Research.

[ref-7] Bianchini E, Calió B, Alborghetti M, Rinaldi D, Hansen C, Vuillerme N, Maetzler W, Pontieri FE (2023). Step-counting accuracy of a commercial smartwatch in mild-to-moderate pd patients and effect of spatiotemporal gait parameters, laterality of symptoms, pharmacological state, and clinical variables. Sensors.

[ref-8] Caroppo A, Manni A, Rescio G, Carluccio AM, Siciliano PA, Leone A (2025). Movement disorders and smart wrist devices: a comprehensive study. Sensors.

[ref-9] Case MA, Burwick HA, Volpp KG, Patel MS (2015). Accuracy of smartphone applications and wearable devices for tracking physical activity data. Jama.

[ref-10] Chan LLY, Lord SR, Brodie MA (2024). Daily-life walking speed, quality and quantity derived from a wrist motion sensor: large-scale normative data for middle-aged and older adults. Sensors.

[ref-11] Danemayer J, Bloomberg M, Mills A, Holloway C, Hussein S (2025). Demographic, socioeconomic, and social barriers to use of mobility assistive products: a multistate analysis of the English longitudinal study of ageing. Lancet Public Health.

[ref-12] Demeyer H, Burtin C, Van Remoortel H, Hornikx M, Langer D, Decramer M, Gosselink R, Janssens W, Troosters T (2014). Standardizing the analysis of physical activity in patients with COPD following a pulmonary rehabilitation program. Chest.

[ref-13] Dixon E, Anderson J, Lazar A (2022). Understanding how sensory changes experienced by individuals with a range of age-related cognitive changes can effect technology use. ACM Transactions on Accessible Computing.

[ref-14] Doherty C, Baldwin M, Keogh A, Caulfield B, Argent R (2024). Keeping pace with wearables: a living umbrella review of systematic reviews evaluating the accuracy of consumer wearable technologies in health measurement. Sports Medicine.

[ref-15] Feehan LM, Geldman J, Sayre EC, Park C, Ezzat AM, Yoo JY, Hamilton CB, Li LC (2018). Accuracy of fitbit devices: systematic review and narrative syntheses of quantitative data. JMIR Mhealth Uhealth.

[ref-16] Garcia FDV, da Cunha MJ, Schuch CP, Schifino GP, Balbinot G, Pagnussat AS (2021). Movement smoothness in chronic post-stroke individuals walking in an outdoor environment—a cross-sectional study using IMU sensors. PLOS ONE.

[ref-17] Holbrook EA, Barreira TV, Kang M (2009). Validity and reliability of Omron pedometers for prescribed and self-paced walking. Medicine and Science in Sports and Exercise.

[ref-18] Holl AE, Spruit MA, Troosters T, Puhan MA, Pepin V, Saey D, McCormack MC, Carlin BW, Sciurba FC, Pitta F, Wanger J, MacIntyre N, Kaminsky DA, Culver BH, Revill SM, Hernandes NA, Andrianopoulos V, Camillo CA, Mitchell KE, Lee AL, Hill CJ, Singh SJ (2014). An official European Respiratory Society/American Thoracic Society technical standard: field walking tests in chronic respiratory disease. European Respiratory Journal.

[ref-19] Holubová A, Malá E, Hoidekrová K, Pětioký J, Ďuriš A, Mužík J (2022). The accuracy of commercially available fitness trackers in patients after stroke. Sensors.

[ref-20] Hulshof CM, Van der Leeden M, Van Netten JJ, Gijssel M, Evers J, Bus SA, Pijnappels M (2024). The association between peripheral neuropathy and daily-life gait quality characteristics in people with diabetes. Gait & Posture.

[ref-21] Keogh A, Argent R, Anderson A, Caulfield B, Johnston W (2021). Assessing the usability of wearable devices to measure gait and physical activity in chronic conditions: a systematic review. Journal of NeuroEngineering and Rehabilitation.

[ref-22] Kobsar D, Masood Z, Khan H, Khalil N, Kiwan MY, Ridd S, Tobis M (2020). Wearable inertial sensors for gait analysis in adults with osteoarthritis—a scoping review. Sensors.

[ref-23] Kooiman TJM, Dontje ML, Sprenger SR, Krijnen WP, van der Schans CP, de Groot M (2015). Reliability and validity of ten consumer activity trackers. BMC Sports Science, Medicine & Rehabilitation.

[ref-24] Kooner P, Schubert T, Howard JL, Lanting BA, Teeter MG, Vasarhelyi EM (2021). Evaluation of the effect of gait aids, such as canes, crutches, and walkers, on the accuracy of step counters in healthy individuals. Orthopedic Research and Reviews.

[ref-25] Kriara L, Dondelinger F, Capezzuto L, Bernasconi C, Lipsmeier F, Galati A, Lindemann M (2025). Investigating measurement equivalence of smartphone sensor-based assessments: remote, digital, bring-your-own-device study. Journal of Medical Internet Research.

[ref-26] Laffi A, Persiani M, Piras A, Meoni A, Raffi M (2025). Effectiveness of wearable technologies in supporting physical activity and metabolic health in adults with type 2 diabetes: a systematic–narrative hybrid review. Healthcare.

[ref-27] Masoumian Hosseini M, Masoumian Hosseini ST, Qayumi K, Hosseinzadeh S, SSajadi Tabar S (2023). Smartwatches in healthcare medicine: assistance and monitoring; a scoping review. BMC Medical Informatics and Decision Making.

[ref-28] Moore K, O’Shea E, Kenny L, Barton J, Tedesco S, Sica M, Crowe C, Alamäki A, Condell J, Nordström A, Timmons S (2021). Older adults’ experiences with using wearable devices: qualitative systematic review and meta-synthesis. JMIR Mhealth Uhealth.

[ref-29] Pan J, Wei S (2024). Accuracy and reliability of accelerometer-based pedometers in step counts during walking, running, and stair climbing in different locations of attachment. Scientific Reports.

[ref-30] Pepera GK, Sandercock GR, Sloan R, Cleland JJ, Ingle L, Clark AL (2012). Influence of step length on 6-minute walk test performance in patients with chronic heart failure. Physiotherapy.

[ref-31] Puterman E, Pauly T, Ruissen G, Nelson B, Faulkner G (2021). Move more, move better: a narrative review of wearable technologies and their application to precision health. Health Psychology.

[ref-32] Schmidle S, Gulde P, Koster R, Soaz C, Hermsdörfer J (2023). The relationship between self-reported physical frailty and sensor-based physical activity measures in older adults—a multicentric cross-sectional study. BMC Geriatrics.

[ref-33] Schumann M, Doherty C (2024). Bridging gaps in wearable technology for exercise and health professionals: a brief review. International Journal of Sports Medicine.

[ref-34] Shiwani MA, Chico TJA, Ciravegna F, Mihaylova L (2023). Continuous monitoring of health and mobility indicators in patients with cardiovascular disease: a review of recent technologies. Sensors.

[ref-35] Singh B, Chastin S, Miatke A, Curtis R, Dumuid D, Brinsley J, Ferguson T, Szeto K, Simpson C, Eglitis E, Willems I, Maher C (2024). Real-world accuracy of wearable activity trackers for detecting medical conditions: systematic review and meta-analysis. JMIR Mhealth Uhealth.

[ref-36] Spartano NL, Zhang Y, Liu C, Chernofsky A, Lin H, Trinquart L, Borrelli B, Pathiravasan CH, Kheterpal V, Nowak C, Vasan RS, Benjamin EJ, McManus DD, Murabito JM (2024). Agreement between apple watch and actical step counts in a community setting: cross-sectional investigation from the Framingham Heart Study. JMIR Biomedical Engineering.

[ref-37] Storm FA, Heller BW, Mazzà C (2015). Step detection and activity recognition accuracy of seven physical activity monitors. PLOS ONE.

[ref-38] Straiton N, Alharbi M, Bauman A, Neubeck L, Gullick J, Bhindi R, Gallagher R (2018). The validity and reliability of consumer-grade activity trackers in older, community-dwelling adults: a systematic review. Maturitas.

[ref-39] Sun Y, Chen J, Ji M, Li X (2025). Wearable technologies for health promotion and disease prevention in older adults: systematic scoping review and evidence map. Journal of Medical Internet Research.

[ref-40] Szeto K, Arnold J, Maher C (2024). The Wearable Activity Tracker Checklist for Healthcare (WATCH): a 12-point guide for the implementation of wearable activity trackers in healthcare. International Journal of Behavioral Nutrition and Physical Activity.

[ref-41] Tedesco S, Barton J, O’Flynn B (2017). A review of activity trackers for senior citizens: research perspectives, commercial landscape and the role of the insurance industry. Sensors.

[ref-42] Vavasour G, Giggins OM, Flood MW, Doyle J, Doheny E, Kelly D (2023). Waist—What? Can a single sensor positioned at the waist detect parameters of gait at a speed and distance reflective of older adults’ activity?. PLOS ONE.

[ref-43] Zeng J, Lin S, Li Z, Sun R, Yu X, Lian X, Zhao Y, Ji X, Zheng Z (2024). Association between gait video information and general cardiovascular diseases: a prospective cross-sectional study. European Heart Journal—Digital Health.

