# Supplementary figures and images for: Why your smartwatch may be misleading your doctor: a cross-sectional study on the impact of mobility aids on wearable accuracy in older adults
Source: PeerJ. 2026 Apr 15;14:e20690. doi: 10.7717/peerj.20690 (PMC13091583; doi:10.7717/peerj.20690)

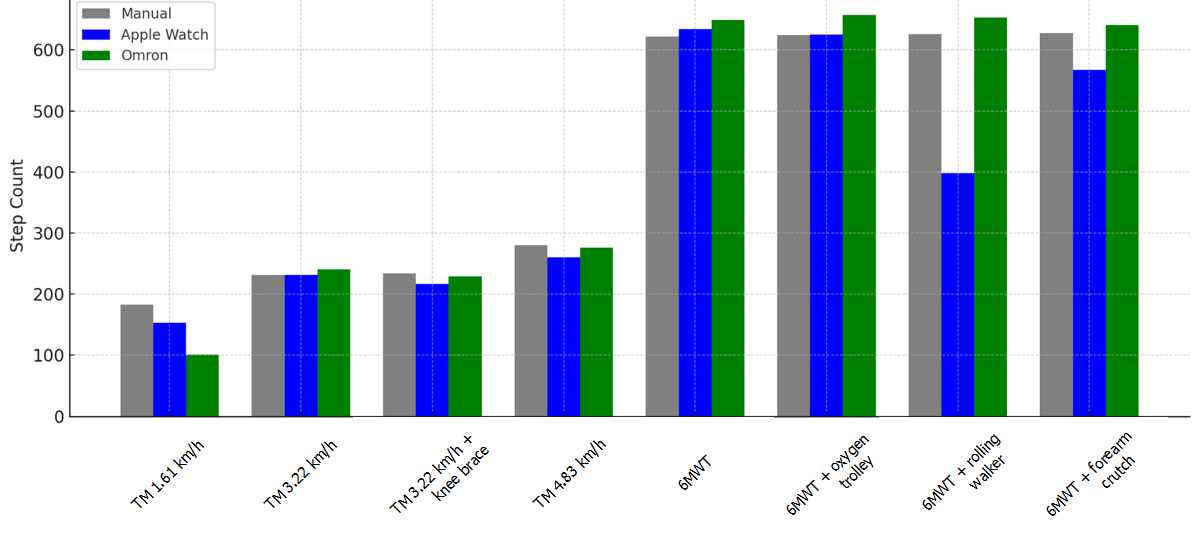

Supplement: Supplemental Information 1 — Compares step counts recorded manually (gray), by the Apple Watch Series 8 (blue), and the Omron Walking Style IV pedometer (green) across various walking conditions. These include treadmill (TM) walking at 1–3 mph, TM with knee brace, and six-minute walk tests (6MWT) with and without assistive devices (oxygen trolley, walker, and forearm crutch). The Apple Watch consistently underestimated steps during assisted walking, especially with the walker, while the Omron pedometer remained closer to manual counts across all conditions. [file peerj-14-20690-s001.png]

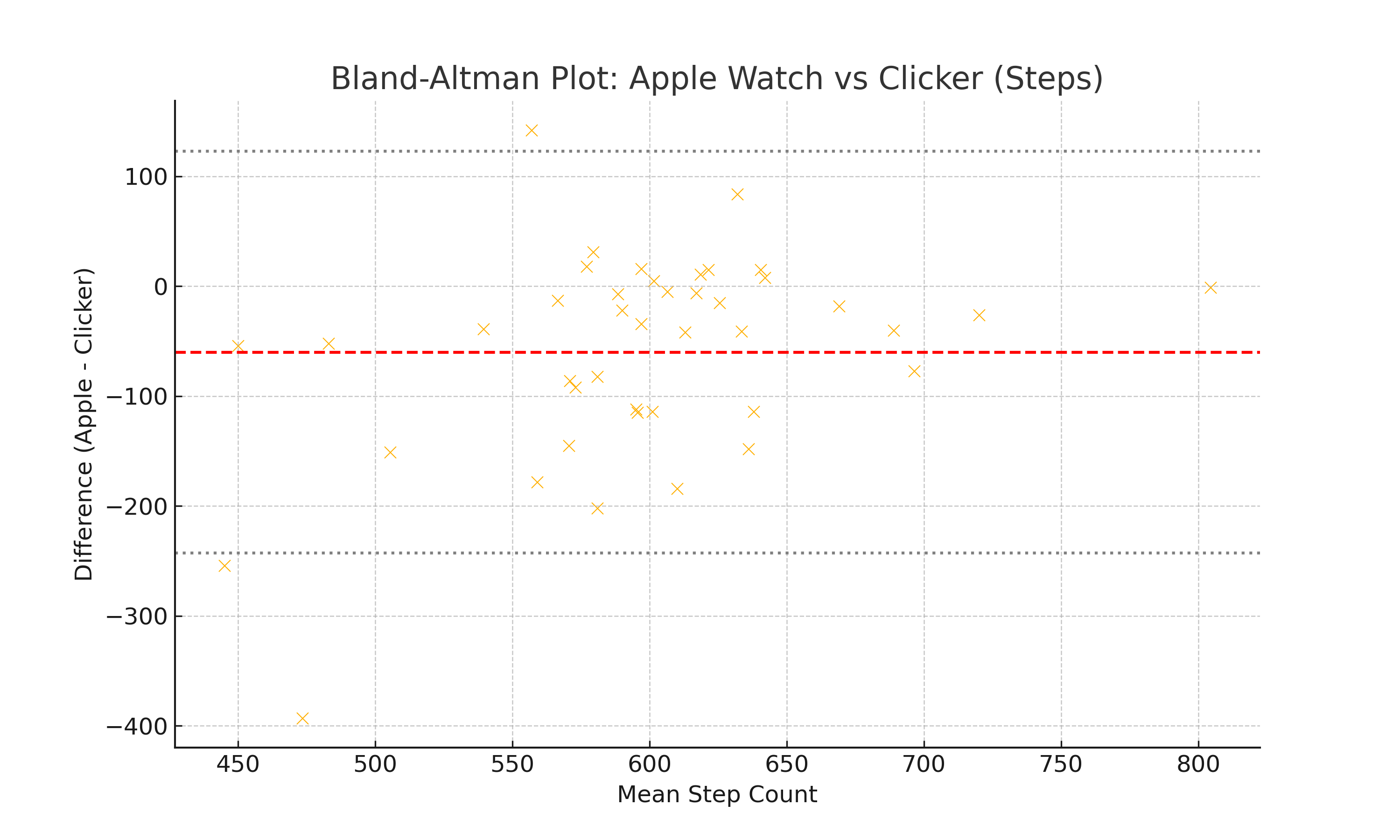

Supplement: Supplemental Information 2 — Illustrates the agreement between step counts recorded by the Apple Watch Series 8 and manual step counts (clicker method). The x-axis represents the mean step count between the two methods, while the y-axis shows the difference (Apple –Manual). The red dashed line indicates the mean bias, suggesting a systematic underestimation by the Apple Watch. Dotted lines represent the 95% limits of agreement. The plot demonstrates moderate variability and consistent undercounting by the Apple Watch across the step count range. [file peerj-14-20690-s002.png]

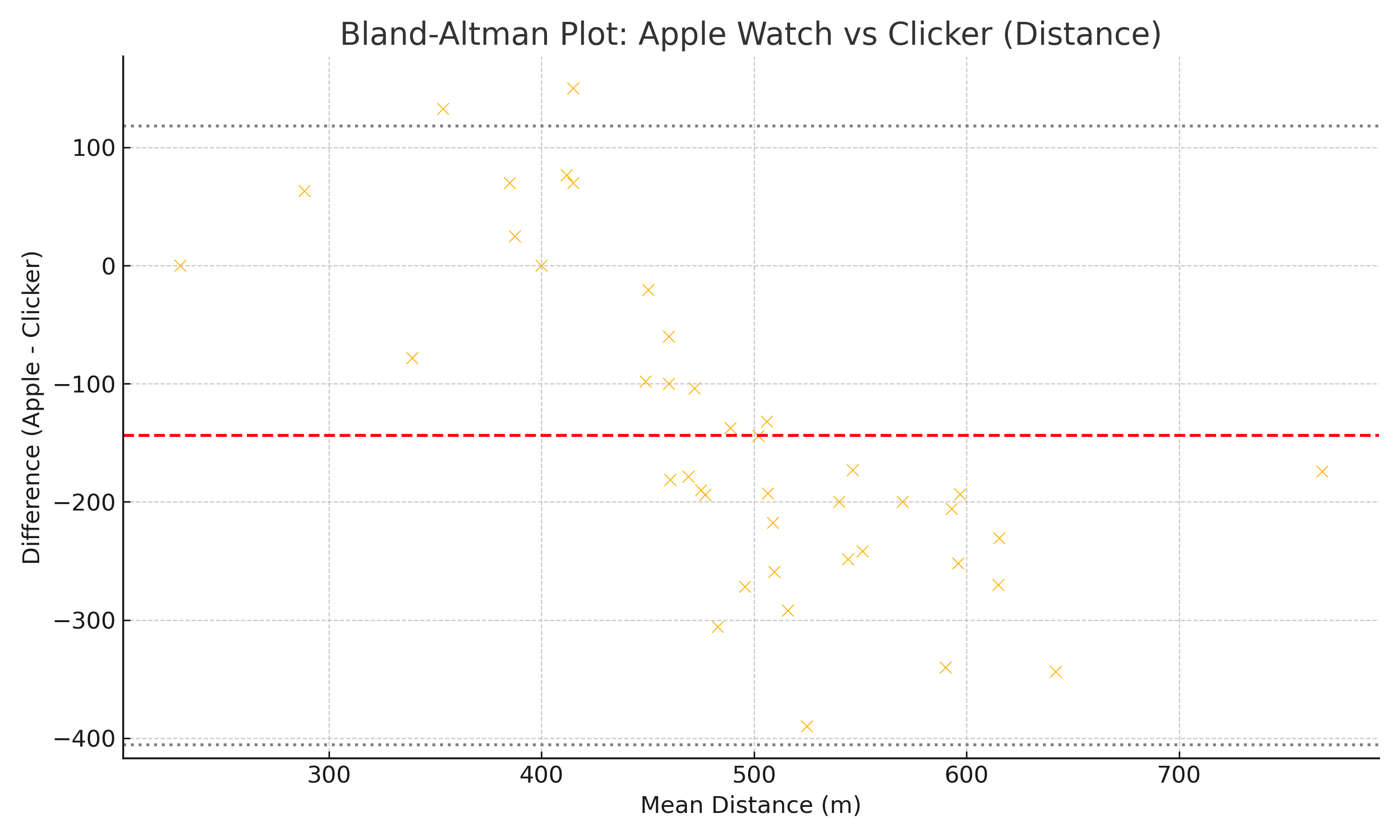

Supplement: Supplemental Information 3 — Compares distance measurements obtained from the Apple Watch Series 8 with manually calculated reference values based on actual walking distance. The x-axis shows the mean distance (in meters) between the two methods, while the y-axis displays the difference (Apple –Manual). The red dashed line represents the mean bias, indicating a consistent underestimation of walking distance by the Apple Watch. Dotted lines indicate the 95% limits of agreement. The plot reveals increased variability in distance estimates at higher walking distances and confirms the Apple Watch’s reduced accuracy in mobility-impaired conditions. [file peerj-14-20690-s003.png]
