# Supplementary material for: Why your smartwatch may be misleading your doctor: a cross-sectional study on the impact of mobility aids on wearable accuracy in older adults
Source: PeerJ. 2026 Apr 15;14:e20690. doi: 10.7717/peerj.20690 (PMC13091583; doi:10.7717/peerj.20690)
Supplement: Supplemental Information 4 [file peerj-14-20690-s004.docx]

**Supplementary Table 1**

Distance Measurements, Distribution, and Percentage Error of Wearable Devices Compared to Manual Measurement Across All Walking Conditions

| **Walking condition** | **Manual distance (km)** | **Apple mean (km)** | **Apple SD** | **Apple median** | **Apple range** | **Apple error (%)** | **Omron mean (km)** | **Omron SD** | **Omron median** | **Omron range** | **Omron error (%)** |
| --- | --- | --- | --- | --- | --- | --- | --- | --- | --- | --- | --- |
| Treadmill 1.61 km/h | 0.050 | 0.114 | 0.009 | 0.10 | 0-0.53 | +128.0% | 0.052 | 0.067 | 0.00 | 0-0.2 | +4.0% |
| Treadmill 3.22 km/h | 0.110 | 0.172 | 0.099 | 0.16 | 0-0.72 | +56.4% | 0.152 | 0.055 | 0.15 | 0.1-0.3 | +38.2% |
| Treadmill 3.22 km/h + knee brace | 0.110 | 0.155 | 0.044 | 0.15 | 0-0.24 | +40.9% | 0.164 | 0.048 | 0.20 | 0.1-0.2 | +49.1% |
| Treadmill 4.83 km/h | 0.170 | 0.211 | 0.090 | 0.20 | 0.1-0.6 | +24.1% | 0.185 | 0.052 | 0.20 | 0.1-0.4 | +8.8% |
| 6MWT | 0.580 | 0.478 | 0.092 | 0.49 | 0.28-0.66 | -17.6% | 0.457 | 0.070 | 0.50 | 0.30-0.60 | -21.2% |
| 6MWT + oxygen trolley | 0.575 | 0.468 | 0.107 | 0.46 | 0.25-0.89 | -18.6% | 0.464 | 0.098 | 0.50 | 0.20-0.80 | -19.3% |
| 6MWT + rolling walker | 0.573 | 0.291 | 0.131 | 0.31 | 0-0.58 | -49.2% | 0.447 | 0.073 | 0.50 | 0.30-0.60 | -22.0% |
| 6MWT + forearm crutch | 0.562 | 0.419 | 0.070 | 0.42 | 0.23-0.68 | -25.4% | 0.442 | 0.066 | 0.40 | 0.30-0.70 | -21.4% |

Note.

^a^Manual distance was measured using treadmill calibration or a 30-meter corridor during the 6-minute walk test (6MWT). Percentage errors reflect the difference between device-reported and manually measured distances.
