# Supplementary material for: Why your smartwatch may be misleading your doctor: a cross-sectional study on the impact of mobility aids on wearable accuracy in older adults
Source: PeerJ. 2026 Apr 15;14:e20690. doi: 10.7717/peerj.20690 (PMC13091583; doi:10.7717/peerj.20690)
Supplement: Supplemental Information 5 [file peerj-14-20690-s005.docx]

**Supplementary Table 2**
Spearman Correlation Matrix Between Participant Characteristics and Device Error Rates

| **Variable** | **1** | **2** | **3** | **4** | **5** | **6** | **7** | **8** | **9** |
| --- | --- | --- | --- | --- | --- | --- | --- | --- | --- |
| 1. Age (years) | 1.00 |  |  |  |  |  |  |  |  |
| 2. Weight (kg) | .18 | 1.00 |  |  |  |  |  |  |  |
| 3. Height (cm) | -.09 | .50** | 1.00 |  |  |  |  |  |  |
| 4. BMI (kg/m²) | .33* | .79** | .09 | 1.00 |  |  |  |  |  |
| 5. Arm length (cm) | -.06 | .68** | .73** | .24 | 1.00 |  |  |  |  |
| 6. Step Error Apple (%) | .06 | .01 | -.12 | .07 | -.12 | 1.00 |  |  |  |
| 7. Step Error Omron (%) | -.05 | -.11 | -.08 | -.08 | -.03 | .11 | 1.00 |  |  |
| 8. Distance Error Apple (%) | .05 | .02 | -.11 | .11 | -.11 | .69** | .33* | 1.00 |  |
| 9. Distance Error Omron (%) | -.02 | -.15 | -.14 | -.09 | -.14 | .30 | .78** | .36* | 1.00 |

Note.

N = 42. Values are Spearman's rho correlation coefficients.

*p < .05, ** p < .01
